# Factors affecting the labelling accuracy of brain MRI studies relevant for deep learning abnormality detection

**DOI:** 10.3389/fradi.2023.1251825

**Published:** 2023-11-27

**Authors:** Matthew Benger, David A. Wood, Sina Kafiabadi, Aisha Al Busaidi, Emily Guilhem, Jeremy Lynch, Matthew Townend, Antanas Montvila, Juveria Siddiqui, Naveen Gadapa, Gareth Barker, Sebastian Ourselin, James H. Cole, Thomas C. Booth

**Affiliations:** ^1^ Department of Neuroradiology, Kings College Hospital, London, United Kingdom; ^2^ School of Biomedical Engineering & Imaging Sciences, Kings College London, London, United Kingdom; ^3^ Institute of Psychiatry, Psychology & Neuroscience, Kings College London, London, United Kingdom; ^4^ Centre for Medical Image Computing, Dementia Research, University College London, London, United Kingdom

**Keywords:** deep learning, computer vision system, labelling, neuroradiology, MRI

## Abstract

Unlocking the vast potential of deep learning-based computer vision classification systems necessitates large data sets for model training. Natural Language Processing (NLP)—involving automation of dataset labelling—represents a potential avenue to achieve this. However, many aspects of NLP for dataset labelling remain unvalidated. Expert radiologists manually labelled over 5,000 MRI head reports in order to develop a deep learning-based neuroradiology NLP report classifier. Our results demonstrate that binary labels (normal vs. abnormal) showed high rates of accuracy, even when only two MRI sequences (T2-weighted and those based on diffusion weighted imaging) were employed as opposed to all sequences in an examination. Meanwhile, the accuracy of more specific labelling for multiple disease categories was variable and dependent on the category. Finally, resultant model performance was shown to be dependent on the expertise of the original labeller, with worse performance seen with non-expert vs. expert labellers.

## 1. Introduction

Deep learning-based computer vision systems hold enormous promise in a variety of clinical applications (1–3) including neuroradiology (4, 5). For example, by enabling appropriate triage of neuroradiology studies, studies with abnormal findings can be flagged for rapid clinical reporting (6–10).

One stumbling block for clinical development is a lack of large clinically representative datasets available to train models. Supervised deep learning models require a labelled dataset, which itself normally requires significant expertise and person-hours for manual labelling. One potential solution to increase the size of datasets is to consider training and using non-expert labellers, which may represent a less expensive and more abundant resource than expert labellers (11). There are various studies in the literature which have investigated the use of “crowdsourcing” to label images as part of non-clinical (12) and clinical computer vision tasks (13, 14). However, no study has investigated whether such crowdsourcing engenders a consequent decrease in labelling performance. We therefore investigated whether using non-expert labellers led to a reduction in labelling accuracy.

Natural Language Processing (NLP) is a further potential avenue to obviate this issue, using text classification models to automatically label MRI studies based on information derived from the original report. NLP necessarily involves classification to transduce written reports into discrete categories. These may be single category (e.g., normal vs. abnormal) or multiple disease categories with varying degrees of granularity. In order to truly validate a single or multi-category classification system for use with NLP, the reports themselves should faithfully reflect the source images. In other words, the report needs to be entirely accurate.

Numerous neuroradiology-focused studies have employed multi-category NLP models as part of computer vision model training (15–17). However, none of these studies provided source image validation of the original scan reports. This study aimed to examine whether the assumption that reports faithfully reflect source imaging is valid in both single category and multi-category classification systems at a UK neurosciences centre. It therefore serves as a demonstration as to how a pragmatic research study may approach label validation.

## 2. Materials and methods

After excluding paediatric (<18 years) examinations, the study included 125,556 adult cerebral MRI studies, reported between 2008 and 2019 by expert neuroradiologists (UK consultant grade; US attending equivalent) at King's College Hospital NHS Foundation Trust (KCH), London, UK. Each report included basic clinical information about the patient and the reasons for the study request, along with a radiological description and conclusion. The UK National Health Research Authority and Research Ethics Committee approved the study.

Prior to labelling, a complete set of 12 clinically relevant neurological disease categories was developed by the research group (a group which included several expert neuroradiologists). In principle, these disease categories were designed to subcategorise the entire gamut of radiologically-relevant cerebral (i.e., non-spinal) neurological diseases and are as follows: supratentorial atrophy, infratentorial atrophy, intracranial mass lesion, extracranial lesion, demyelinating disease, acute infarction, chronic damage, vasculopathy, small vessel disease, foreign body, haemorrhage and hydrocephalus. Of the 125,556 neuroradiology MRI reports available for the study, 5,000 were randomly obtained and subsequently labelled consecutively by expert neuroradiologists in order to train the neuroradiology report classifier [Automated Labelling using an Attention model for Radiology reports of MRI scans (ALARM) classifier], the details of which have been previously described in the following conference proceeding (16). Five thousand was considered a large training dataset likely to capture most examples of radiological disease, almost all examples of radiological normal, and together highly likely to capture the lexical report content which would describe something as normal or abnormal (16). All labellers were blinded to one another except at consensus as described below.

Of the 5,000, 3,000 were independently labelled by 2 expert neuroradiologists and were categorised as abnormal (i.e., containing at least one of the clinically relevant conditions) or normal. For this single category labelling task, the reported agreement value was 94.9%. Then, for the discrepant labels, a third neuroradiologist was employed to generate a consensus outcome to complete a labelled report dataset.

The remaining 2,000 reports were independently labelled by 3 expert neuroradiologists and more specifically classified as to whether they demonstrated any of 12 pre-determined categories of neuroradiological abnormalities. Complete agreements amongst the 3 neuroradiologists occurred in 95.3% of cases, with a consensus decision being made for the remainder to complete a labelled report dataset. The total of 5,000 labels generated in this fashion by neuroradiology consultants is henceforth referred to as “silver standard” reference labels for the corresponding images.

Expert neuroradiologists are a limited resource and an order of magnitude less frequent than either neurologists or junior radiologists (UK registrar grade; US resident equivalent) who might be considered the most optimal of all non-expert labellers. Therefore, the study group included both a non-expert physician (neurologist with 10 years clinical experience involving brain MRIs) and a junior radiologist (with 3 years of general radiology training). After a 6-month period of label training, both also labelled the data in an identical fashion to above. All labellers joined fortnightly team meetings over the category development phase which lasted 6 months. Training took place based on category consensus as well as edge case discussions. The discussions were often preceded by discrepancies and so the process served as a form of formative assessment. The performance of the ALARM classifier trained using the individual physician-derived labels and the individual junior radiologist-derived labels was compared to the performance of a model trained using individual neuroradiologist-derived labels. The F1-score—a harmonic mean of precision and recall—was used to quantify model performance.

A set of 500 labels was generated from source image analysis primarily to perform checks on the NLP label process i.e., the MRI studies themselves were re-assessed and labelled by the expert neuroradiologist, as opposed to the reports. The set was randomly selected. The details of the labelling process itself is similar to “silver standard” reference labels, with 250 studies being categorised as normal vs. abnormal (by 2 neuroradiologists with a third neuroradiologist employed to generate a consensus outcome for discrepant labels) and 250 studies being labelled on the presence or absence of 12 categories of neuroradiological abnormalities (also by consensus based on 3 neuroradiologists). Labels generated in this manner are henceforth referred to as “gold standard” reference labels for the corresponding images. A qualitative assessment of “silver standard” and “gold standard” label discrepancies was carried out by our group of expert neuroradiologists. We examined whether an error appeared to be due to a deliberate omission by the reporting radiologist, or whether the omission was accidental.

## 3. Results

### 3.1. Comparing performance of non-expert vs. expert labelling

**Figure 1:**
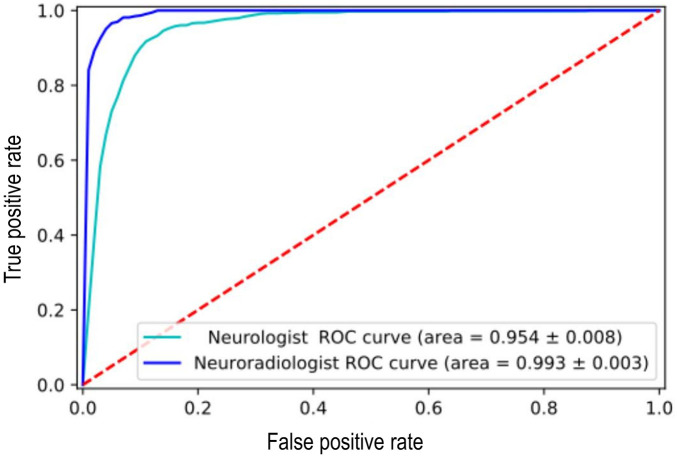
Reduction in [Automated labelling using an attention model for radiology (ALARM)] classifier model performance when it was trained using the non-expert labels vs. expert labels at an arbitrarily fixed sensitivity of 90%.

The ALARM classifier model was trained using both single category (normal vs. abnormal) individual physician-derived labels and individual expert neuroradiologist-derived labels and then tested on a set of silver standard reference labels. There was a significant reduction in model performance accuracy when the model was trained using the physician's labels (F1 score = 0.76) compared to the neuroradiologist's labels (F1 score = 0.90) (Figure 1). Given the low performance accuracy based on physician-derived labels in the single category labelling task, a non-radiologist labelling strategy was not pursued as a potential alternative to neuroradiologist labelling in the multicategory labelling task. In the multicategory labelling task, a junior radiologist labelling strategy was examined. However, there was a reduction in ALARM classifier performance accuracy when junior radiologist-derived labels were used (F1 score = 0.71).

### 3.2. Report validation

We next compared the performance of our silver standard reference (consensus labelling based on reports) to our gold standard reference (consensus labelling based on re-review of the corresponding MRI source images).

The sensitivity of the silver standard reference labels varied amongst the 12 categories of abnormality. In particular, haemorrhage (including microhaemorrhage), hydrocephalus (including chronic), extracranial abnormality, and infratentorial atrophy demonstrated a sensitivity below 80% whilst certain categories including infarct, foreign body, mass, small vessel disease, white matter inflammation and supratentorial atrophy had a sensitivity above 90% (Table 1). Importantly, the silver standard reference labelling system had an excellent sensitivity (98.7%), specificity (96.6%) and accuracy (98.5%) regarding separating normal and abnormal studies using the gold standard reference.

**Table 1:** Sensitivity, specificity and F1-score of the silver standard reference labels amongst the 12 categories of abnormalities.

Category	Sensitivity (%)	Specificity (%)	F1 score (%)
Foreign Body	100	99.6	99.6
Supratentorial atrophy	100	94.6	76.9
Intracranial mass lesion	97.9	93.6	95.9
Demyelinating disease	95.6	100	97.7
Acute infarction	94.4	99.5	94.4
Small vessel disease	90.5	95.6	93.2
Vasculopathy	83.3	88.4	86.5
Chronic damage	82.4	92.7	87.8
Infratentorial atrophy	77.7	94.3	54.5
Hydrocephalus	70.0	99.6	77.8
Haemorrhage	69.2	99.6	78.3
Extracranial lesion	60.0	94.7	54.5
Mean	85.1	96.0	82.8

### 3.3. Sequence-specific performance

**Figure 2:**
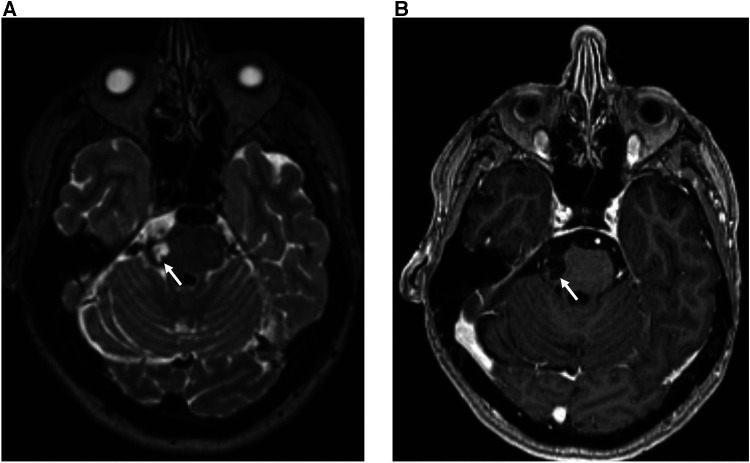
30 year-old female with sudden onset headache and right-sided facial sensory disturbance. MRI head study performed several days after initial symptom onset demonstrates a subacute haematoma on the lateral aspect of the right ventral pons at the site of entry of the right trigeminal nerve characterized by: (**A**) high T2 signal relative to grey matter on the axial T2-weighted sequence (white arrow) (**B**) low T1 signal relative to grey matter and absence of contrast enhancement on the gadolinium-enhanced axial T1-weighted sequence (blue arrow). The patient was subsequently found to have a small arteriovenous malformation on digitally-subtracted cerebral angiography (not shown).

An MRI report consists of a single assessment of multiple imaging sequences, with each sequence providing different tissue-specific information that is synthesised by the neuroradiologist to produce a cohesive answer to the clinical question. For example, multiple imaging sequences are required to estimate chronicity: haemorrhage signal intensity changes over time eventually becoming dark on both T1-weighted and T2-weighted images after ∼30 days (Figure 2). Broadly speaking, an expert neuroradiologist (and potentially a computer vision system), using limited sequences only for labelling, loses information if all the multiple input sequences are not used for a single examination-level label. For example, in the case of haemorrhage, a lack of T1-weighted images would engender a loss of information regarding haemorrhage chronicity. This would be important information loss if a cohesive answer to the clinical question were required, for example in a diagnostic task. Conversely, if the only information required was the presence or absence of an abnormality, such as in a triage task, we hypothesized that a limited number of sequences would provide sufficient information. In Figure 2, for example, either T1-weighted or T2-weighted images would suffice.

**Figure 3:**
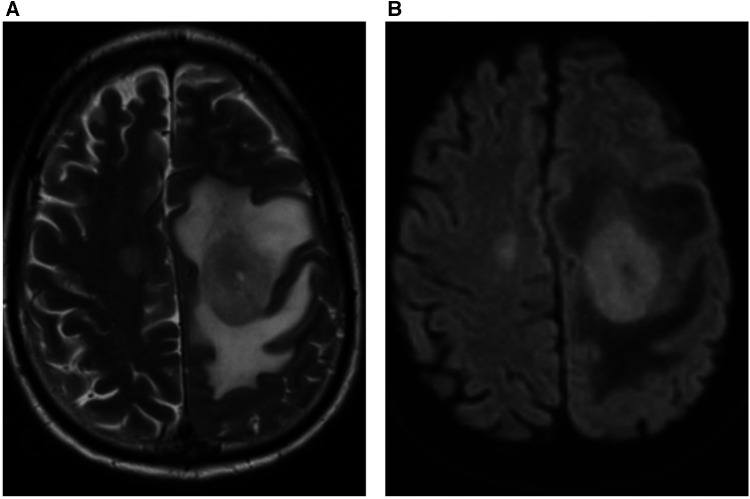
59 year-old male patient presented with right arm and leg weakness. (**A**) Axial T2-weighted MRI head sequence demonstrates an ovoid lesion in the left centrum semiovale which is hyperintense relative to grey matter. There are features consistent with surrounding peri-lesional vasogenic oedema. There is a further smaller lesion in the right centrum semiovale. (**B**) Both lesions demonstrate homogenous, increased diffusion restriction relative to background brain parenchyma on the axial DWI MRI head sequence. The patient was diagnosed with primary cerebral lymphoma based on imaging features and cerebrospinal fluid (CSF) analysis.

Therefore, we investigated whether clinically-useful information could be derived from a minimal number of frequently-used MRI sequences. Specifically, we chose standard axial T2-weighted sequences and those based on diffusion weighted imaging (DWI), which are used together in over 80% of MRI studies at our institution. We investigated the percentage of abnormal findings detectable using only these sequences. Single binary labelling (normal or abnormal) was performed by an expert neuroradiologist looking at only the T2-weighted and DWI information for 250 studies and this was compared to binary labelling derived from all available sequences generated by a different expert neuroradiologist. Inter-observer agreement was 98%, demonstrating the utility of using these sequences alone in defining studies as normal or abnormal. An extremely wide range of abnormalities were detected using only T2-weighted and DWI information. An example of cerebral lymphoma detected as “abnormal” using limited sequence information is shown in Figure 3.

## 4. Discussion

Accurate dataset labelling is a crucial step in the development of optimal computer vision classification systems. This was the first research study to investigate the accuracy of this labelling process itself.

One crucial category of dataset labelling is accurately differentiating normal and abnormal studies. This facilitates the development of computer vision classification systems that can identify and triage “abnormal” studies for urgent radiology reporting (18). Reassuringly, comparing our silver standard reference labels to our gold standard reference, the accuracy of expert neuroradiologists was found to be very high.

Interestingly, the accuracy of labelling dropped off significantly when performed by non-expert labellers who had relevant clinical experience and had undergone considerable labelling preparation before the task. Specifically, the F1-score decreased from 0.90 to 0.76. The ramifications of this lower F1-score need to be considered. The F1-score is the harmonic mean of the model's precision (number of true positive predictions divided by the total number of positive predictions) and recall (number of true positive predictions divided by the total number of actual positive cases) (19). In many domains, an F1-score of >0.75 is considered acceptable. However, in neuroimaging, a higher threshold is required. Specifically, in a theoretical “triage-based” Artificial Intelligence (AI) clinical pathway, a false-negative AI report (engendered by a low recall score for the model) may place the incorrectly-reported study to the bottom of the queue for human reporting and potentially significantly delay diagnosis. The exact definition of a “good enough” F1-score for neuroimaging applications remains to be determined and indeed, at the time of writing, almost no neuroimaging abnormality detection tools have been adequately validated in representative clinical cohorts (10), although there are a few notable exceptions (7, 8).

Despite the shortage of expert neuroradiologists as labellers, our research suggests that employing experts in the labelling process of brain MRIs is necessary to ensure labelling accuracy and corresponding performance accuracy of any computer vision classification system derived from it. It is worth noting that an alternative strategy, employing semi-supervised training with limited labelled data, has recently been proposed in the field of chest x-ray imaging (20), however limited data are currently available and no equivalent research has yet been performed in the field of neuroimaging.

The degree of discrepancy between labellers of different clinical backgrounds and experience is notable. Across all institutions, the reporting style of expert neuroradiologists is variable with certain authors tending towards greater complexity (for example, more detailed anatomical and sequence-specific descriptions) while others tend towards a more broad-brush and succinct style. It is plausible that the former reporting style may not lend itself as easily to interpretation and labelling by non-experts. For example, the phrase “there are multiple foci of SWI signal drop out indicating areas of haemosiderin deposition” may be less likely to yield a correct label than the synonymous phrase “there are multiple microhaemorrhages”. Although, clearly, there is no “correct” reporting style, the drop-off in labelling accuracy by non-experts might demonstrate the potential for report misinterpretation by those less well acquainted with the neuroradiological lexicon, and hence the importance of tailoring one's report to the target audience.

The accuracy of labelling for disease categories of pathology was variable and dependent on differing sensitivity of radiology reports for each category (i.e., inaccurate disease categories were ones which were less frequently remarked upon in the report when present on the image as opposed to incorrect attribution to another disease category).

A discussion amongst our group of expert neuroradiologists established likely causes for this. Firstly, when writing radiology reports, radiologists often exclude mentioning pathologies that bear no relevance to the clinical question at hand. For example, benign extracranial abnormalities and chronic atrophic brain changes may not be relevant when reporting a follow-up scan for a malignant brain tumour resection. Similarly, one or two microhaemorrhages are a common finding in ageing brains and frequently of minimal clinical relevance, therefore some neuroradiologists may not choose to mention them (21, 22). Furthermore, post-operative hydrocephalus, if present pre-operatively and reducing in volume over time, may be an entirely expected finding and the report might be worded to ensure there is no miscommunication which could otherwise trigger an unnecessary urgent intervention. In contrast, a post-operative infarct or a residual brain tumour mass are almost always of high clinical relevance and therefore are likely to have a higher comparative sensitivity within a report.

A second source of low sensitivity for some granular categories was thought to be related to “satisfaction of search” errors in the reports. In particular, MRI scans are frequently performed to rule out a specific type of pathology—for example a patient with memory loss may have an MRI study to look for supratentorial atrophy in particular. For such studies, neuroradiologists may tailor their search method accordingly, and may therefore have a higher probability of failing to search for additional co-existent and/or incidental pathology, such as extracranial pathology. There is, of course, an overlap with the first cause of low sensitivity, in that certain pathologies may be considered by the reporter to be irrelevant and therefore not searched for in the first place.

Finally, we established the potential utility of limited sequence labelling to overcome potential real-world time issues regarding reviewing multiple input sequences for a single examination-level label. The majority of “clinically-relevant” lesions were identified using T2-weighted and DWI sequences only. However, particular lesions may not be identified by this combination of sequences—for example, although we did not find the following false negatives in the current study, it is conceivable that certain infections and neoplasms may be identified on post-contrast imaging only (23, 24). Similarly, haemorrhage detection is greatly facilitated by a T2*-weighted sequence (25, 26).

A limitation of our study is the potential for institution-dependent bias in reporting, impacting upon the sensitivity weighting of the various categories. Each institution will tend to focus on different patient demographics, different subspecialty areas and may have a different reporting ethos in terms of what pathology they tend to include in reports. This highlights a general problem with single institution labelling.

Another limitation is the use of single labeller comparisons to determine the suitability of the type of labeller (expert or non-expert). Larger numbers of expert and non-expert labellers would be required to comprehensively confirm the plausible inference that the performance of non-expert labellers is inferior to expert labellers and, therefore, that expert labellers are required to label datasets accurately for brain MRI. Even if an experiment was performed with a larger number of non-experts and we found that all new non-experts were not inferior to experts, knowledge that just a few non-experts are inferior would prevent the rational use of non-expert labellers routinely. Furthermore, we had little incentive to explore this further given the considerable resource requirements to confirm inferiority of non-experts definitively. Instead, we recommend that future experiments that require labelling rely upon expert labellers alone.

Finally, whilst this study highlights the efficacy of combining T2-weighted and DWI sequences for labelling MRI scans as “normal” or “abnormal”, it should be noted that these sequences were chosen pragmatically given their relatively frequent incorporation into scanning protocols at King's College Hospital, London. Other combinations of MRI sequences could have been chosen for experimentation: in particular, future experiments may look to combine Fluid Attenuated Inversion Recovery (FLAIR)—which improves detection of lesions adjacent to CSF-containing spaces (27)—and DWI to see if this improves the accuracy of the detection algorithm still further.

In conclusion, we have demonstrated the high performance of the silver standard reference (consensus labelling based on reports) in terms of single category binary labelling of “normal vs. abnormal”. This labelling process is relatively expedient in terms of time taken when compared to the “gold standard” labelling reference standard involving re-reporting of source images. Notably, this research also demonstrates the importance of employing expert neuroradiologists in the labelling process of brain MRIs, given the significant drop in performance observed when labelling was performed by an experienced, but non-expert, physician or a junior radiologist. The silver standard reference labelling demonstrated a more variable performance regarding 12 granular labelling categories, likely secondary to the relatively infrequent inclusion of particular categories in neuroradiology reports. Finally, this research shows that the majority of “clinically-relevant” lesions may be identified as abnormal (vs. normal) using T2-weighted sequences and those based on diffusion weighted imaging only, potentially limiting the number of MRI sequences required to train computer vision systems.

## Data Availability

The raw data supporting the conclusions of this article will be made available by the authors, without undue reservation.
